# Soil Nutrients, Enzyme Activities, and Microbial Communities along a Chronosequence of Chinese Fir Plantations in Subtropical China

**DOI:** 10.3390/plants12101931

**Published:** 2023-05-09

**Authors:** Junjie Lei, Yixuan Cao, Jun Wang, Yazhen Chen, Yuanying Peng, Qiwen Shao, Qing Dan, Yichen Xu, Xiaoyong Chen, Peng Dang, Wende Yan

**Affiliations:** 1 College of Life Science and Technology, Central South University of Forestry and Technology, Changsha 410004, China; 2 National Engineering Laboratory for Applied Technology of Forestry & Ecology in South China, Changsha 410004, China; 3 College of Arts and Sciences, Saint Xavier University, Chicago, IL 60655, USA; 4 College of Arts and Sciences, Governors State University, University Park, IL 60484, USA; 5 College of Forestry, Central South University of Forestry and Technology, Changsha 410004, China

**Keywords:** Chinese fir plantation, ecological stoichiometry, soil enzymes, soil microorganisms, soil nutrients

## Abstract

Forests undergo a long-term development process from young to mature stages, yet the variations in soil nutrients, enzyme activities, microbial diversity, and community composition related to forest ages are still unclear. In this study, the characteristics of soil bacterial and fungal communities with their corresponding soil environmental factors in the young, middle, and mature stages (7, 15, and 25-year-old) of Chinese fir plantations (CFP) in the subtropical region of China were investigated in 2021. Results showed that the alpha diversity indices (Chao1 and Shannon) of soil bacteria and fungi were higher in 15 and 25-year-old stands than in 7-year-old stand of CFP, while the soil pH, soil water content, soil organic carbon, total nitrogen, total phosphorus, sucrase, urease, acid phosphatase, catalase, and microbial biomass carbon, nitrogen, and phosphorus showed higher in 7-year-old stand than other two stands of CFP. The nonmetric multidimensional scaling analysis revealed that the soil microbial species composition was significantly different in three stand ages of CFP. The redundancy and canonical correspondence analysis indicated that the soil urease and microbial biomass nitrogen were the main factors affecting soil bacterial and fungal species composition. Our findings suggested that soil microbial diversity and community structure were inconsistent with changes in soil nutrients and enzyme activities during CFP development, and enhancing stand nurturing and soil nutrient accumulation in the mid-development stage were beneficial to the sustainable management of CFP.

## 1. Introduction

Forest soils are important globally for many reasons, including the relatively large amount of carbon and nutrients stored in forest soil organic matter [1]. Soils provide physical support, supply nutrients and moisture for growth, and store elements for recycling back to trees [2]. Forest soils are influenced by forest vegetation types, vegetation ages, climate, parent material, and other factors [3,4]. Soil carbon (C), nitrogen (N), and phosphorus (P) are the essential components of soil nutrients and are the necessary elements for plant growth, and their stoichiometry relationship directly indicates the nutrient regulation mechanisms between plants and soil [5]. 

As the most active part of the forest ecosystem, microorganisms play a vital role in the formation and decomposition of soil organic matter, material cycle, and energy flow, and are the link between soil and plants [6]. Soil microorganisms were driving factors in the cycle of soil nutrients such as C, N, and P [7]. Among them, soil enzymes, as the main biocatalysts for the decomposition, turnover, and mineralization of organic matter, accelerate the process of the soil nutrient cycle, and their enzyme activity can indicate the nutrient requirements of microorganisms’ relationship with soil nutrient supply [7,8,9]. The diversity and abundance of soil microbial species were affected by soil physical and chemical properties, root exudates, and plant residues, as well as the changes in stand ages can lead to the differences in stand structure, forest microclimate, and litter quantity and quality in forest ecosystems [10,11]. 

Chinese fir (*Cunninghamia lanceolata*) is the main afforestation tree species with over 1000 years of cultivation history and high yield and excellent woody quality characters widely distributed in subtropical regions of China, effectively alleviating the current contradiction between timber supply and demand [12]. Chinese fir plantation (CFP) is covering an area of 9.87 million hectares, with a storage volume of 755 million cubic meters, accounting for 25–30% of the country’s annual commercial timber production [13]. The contribution of CFP to the sustainable development of plantation forests and the global carbon cycle is widely recognized. However, due to the continuous shortening of the crop rotation cycle and long-term monoculture, serious problems such as soil fertility degradation, productivity decline, and species diversity reduction seriously affecting the functions and services of the plantation forest ecosystem were found in CFP [14,15]. 

Numerous studies have confirmed that soil nutrients are significantly negatively correlated with enzyme activity, which mainly depends on the availability of soil nutrients to microorganisms [9,16]. In contrast, it was demonstrated that the increasing input of soil organic matter can significantly enhance the activity of carbon decomposition-related enzymes [17]. The differences in the intra-forest microenvironment, vegetation yield, and canopy density along stand age sequences affect the throughfall, solar radiation, and litter distribution, with a consequent influence on soil organic matter content, enzyme activity, and microbial community characteristics [6,18]. Forest stand age has been found to affect soil nutrient allocation patterns by altering stand structure, material composition, and intra-forest microclimate in plantation forest ecosystems. The previous research results of changes in soil microorganisms of CFP at different stand ages had unclear patterns, such as the ‘low-middle-high’, ‘high-low-high’, and ‘low-high-low’ corresponding patterns with increasing stand ages of CFP [19]. The variation of patterns might be driven by soil environmental factors such as soil pH, total nitrogen, elemental stoichiometry ratio, and enzyme activity [20]. However, soil environmental factors changing arising from the stand development of CFP on soil nutrient contents, the microbial diversity and community structure, and its driving mechanisms are still unclear. It is necessary to understand the relationship between the soil nutrient characteristics and soil microbial community and stand age development in CFP.

This study aimed to investigate the characteristics of changes in soil nutrients, microbial biomass, enzyme activities, and microbial communities and their interrelationships at different developmental stages of CFP in subtropical China. The objectives of this study were (1) to investigate the vertical distribution of soil nutrients, enzyme activities, and microbial biomass in different stand ages of CFP, (2) to explore the variations of soil microbial diversity and community composition in different stand ages of CFP, and (3) to reveal the main soil environmental effect factors of microbial communities in CFP. 

## 2. Results

### 2.1. The Characteristics of Soil Physicochemical Properties, Microbial Biomass, and Enzyme Activities in Stand Ages and Soil Depth

**Figure 1:**
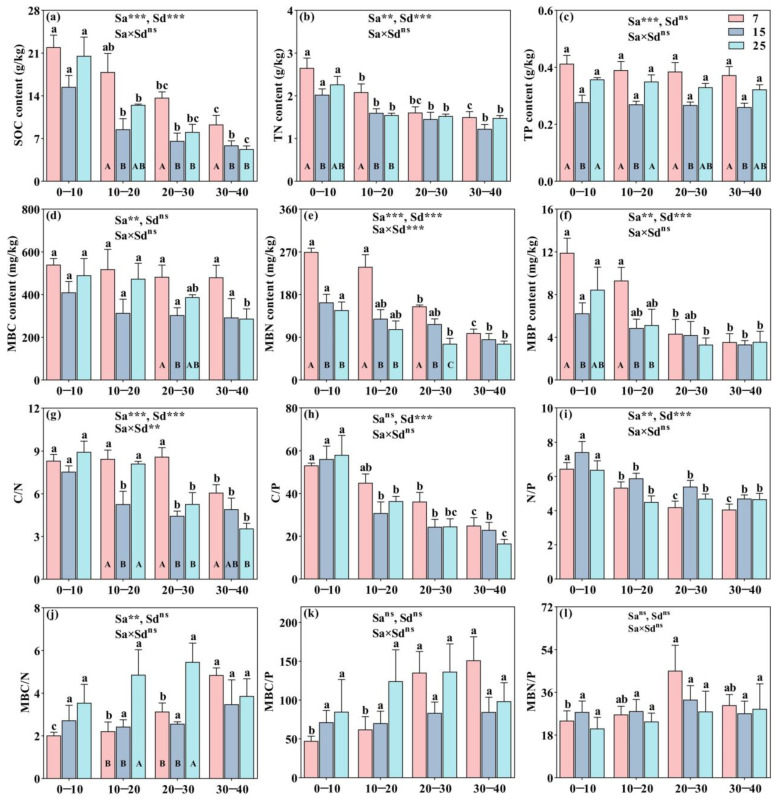
Vertical destitution of soil nutrients and their stoichiometric characteristics in different stand ages of CFP (7, 15, and 25-year-old) was represented in subfigures of (**a**–**f**) and (**g**–**l**), respec-tively. Bar indicates means ± standard error (*n* = 4). Different lowercase and uppercase letters rep-resent the significant difference in soil nutrients and their stoichiometric at different soil depths and in different stand ages of CFP, respectively (Tukey’s honestly significant difference test, *p* < 0.05). ** and *** indicate significance at *p* < 0.01 and *p* < 0.001, respectively, and ns represents no significant differences (*p* > 0.05). SOC is soil organic carbon, TN is soil total nitrogen, TP is soil total phosphorus, MBC is microbial biomass carbon, MBN is microbial biomass nitrogen, MBP is microbial biomass phosphorus, Sa is stand age, Sd is soil depth, and CFP is Chinese fir plantation.

Stand ages significantly affected the soil nutrients and their ecological stoichiometry, including SOC, TN, TP, MBC, MBN, MBP, C/N, N/P, and MBC/N (*p* < 0.01, Figure 1). Except for MBN, all soil nutrient contents of CFP showed a general pattern that 7-year-old > 25-year-old > 15-year-old (Table 1). The stoichiometric of soil C/N, C/P, MBC/N, and MBC/P were lower in 15-year-old stand of CFP than those in 7 and 25-year-old stands, while the C/N in 7-year-old stand, and the MBC/N in 25-year-old stand of CFP were significantly higher than other two stand ages, respectively (*p* < 0.05, Table 1).

Soil depth significantly affected SOC, TN, TP, MBN, MBP, C/N, C/P, and N/P, which showed a decreasing pattern with an increase in soil depth (*p* < 0.01, Figure 1). Whereas, the value of MBC/N, MBC/P, and MBN/P showed a general pattern of gradually increasing in the 0–10, 10–20, and 20–30 cm soil layers (Figure 1j–l). A significant difference was found in SOC, TN, MBN, MBP, C/N, C/P, and N/P between 0–10 and 30–40 cm in different stand ages of CFP (*p* < 0.05, Figure 1).

Moreover, soil MBN and C/N were significantly affected by the interaction of stand age and soil layer (*p* < 0.01, Figure 1e,g). Soil TP had significant variations in response to stand ages at the four soil layers, showing 7-year-old > 25-year-old > 15-year-old (*p* < 0.05, Figure 1c), while C/P, N/P, MBC/P, and MBN/P had no significant variation with stand ages in different soil layers (Figure 1h,i,k,l). Soil TN, MBN, and MBP varied significantly with stand age in the 0–10 and 10–20 cm soil layers, all of which were the highest in 7-year-old stand (*p* < 0.05, Figure 1b,e,f).

**Figure 2:**
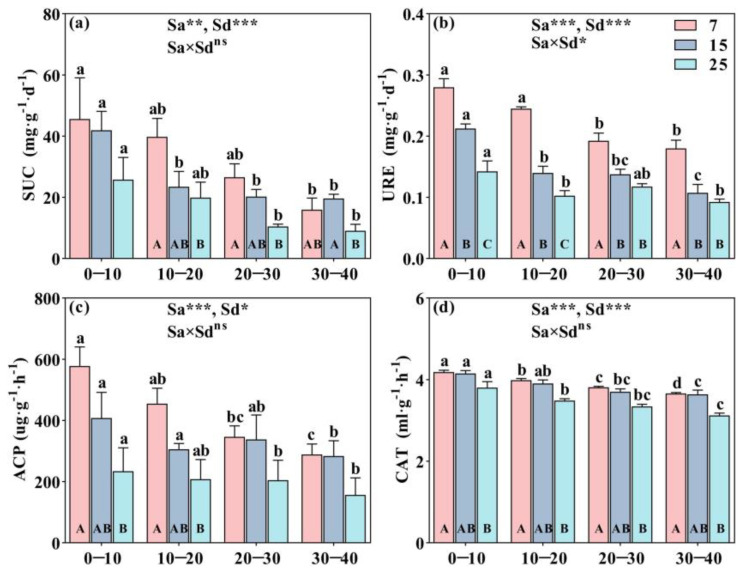
Vertical distribution of soil enzyme (SUC, URE, ACP, and CAT) activities in different stand ages of CFP (7, 15, and 25-year-old) was represented in subfigures (**a**–**d**), respectively. Bar indicates means ± standard error (*n* = 4). Different lowercase and uppercase letters represent the significant difference in soil nutrients and their stoichiometric at different soil depths and in different stand ages of CFP, respectively (Tukey’s honestly significant difference test, *p* < 0.05). *, **, and *** indicate significance at *p* < 0.05, *p* < 0.01, and *p* < 0.001, respectively, and ns represents no significant differences (*p* > 0.05). SUC is Sucrase, URE is Urease, ACP is Acid phosphatase, CAT is Catalase, Sa is stand age, and Sd is soil depth.

Stand ages and soil depth of CFP had significant effects on all soil enzyme activities, which showed that all SUC, URE, ACP, and CAT activities were 7-year-old > 15-year-old > 25-year-old throughout all soil layers (*p* < 0.05, Figure 2), excepting the SUC activity at 30–40 cm soil layer (Figure 2a). The decreasing pattern of enzyme activities as the soil depth increased (Figure 2a–d). URE and CAT had significant variations in response to stand ages at the four soil layers, where URE was significantly affected by the interaction of stand age and soil layer (*p* < 0.05, Figure 2b,d).

### 2.2. Soil Microbial Diversity and Community Composition

**Figure 3:**
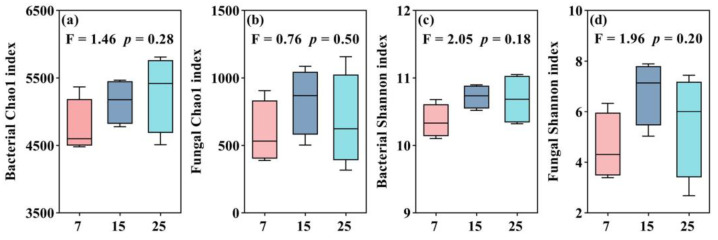
Alpha diversity of soil bacterial and fungal in different stand ages of CFP (7, 15, and 25-year-old). Bacterial and fungal Chao 1 index and Shannon index are represented in subfigures of (**a**–**d**), respectively. Bar indicates means ± standard error (*n* = 4). Differences in bacterial and fungal alpha diversity indices among different stand ages using Tukey’s honestly significant difference test.

The species richness of bacteria gradually increased with the increasing stand ages of CFP on the topsoil (0–10 cm) (Figure 3a). The changes in bacterial diversity were 15-year-old > 25-year-old > 7-year-old stand, and the same pattern of fungal species richness and diversity were found as well in CFP (Figure 3b–d).

**Figure 4:**
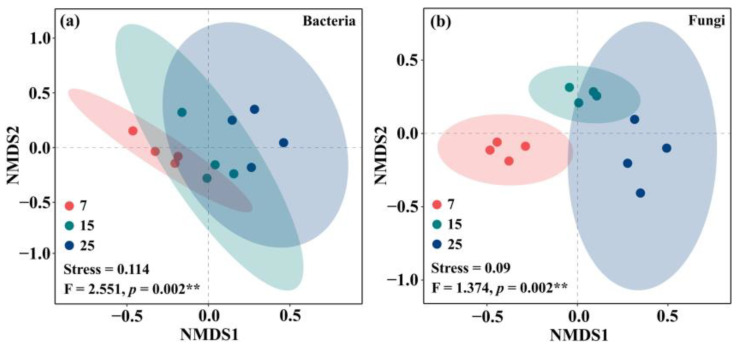
Nonmetric multidimensional scaling ordination plot based on the samples Bray-Curtis distance of bacterial (**a**) and fungal (**b**) communities in different stand ages of CFP (7, 15, and 25-year-old). ** indicate significance at *p* < 0.01.

Stand ages of CFP had significant effects on soil microbial community β-diversity, and the ages of CFP significantly altered bacterial and fungal communities (Stress = 0.114, *p* = 0.002; Stress = 0.09, *p* = 0.002) (Figure 4). The significant differences in soil bacterial communities were found in three developmental stages of CFP (Figure 4a), while the differences in fungal communities were more pronounced between young CFP and the other two stand ages of CFP (Figure 4b).

**Figure 5:**
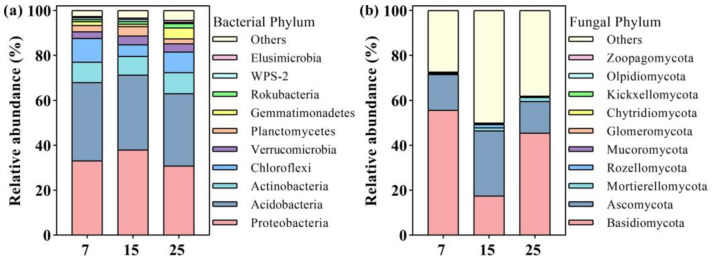
Average relative abundance (%) of bacterial (**a**) and fungal (**b**) communities at the phylum level (*n* = 10) in different stand ages of CFP (7, 15, and 25-year-old).

The compositions of soil bacterial and fungal communities in different stand ages of CFP was shown in Figure 5. The composition of bacterial communities was dominated by species of *Proteobacterial*, *Acidobacterial*, *Actinobacteria*, and *Chloroflexi*, accounting for 87.6–81.5% of the total sequence with the remaining species of *Verrucomicrobia*, *Planctomycetes*, *Gemmatimonadetes*, *Rokubacteria*, *WPS-2*, and *Elusimicrobia* (Figure 5a). The composition of fungi communities was dominated by phyla of *Basidiomycota*, *Ascomycota*, *Mortierellomycota*, and *Rozellomycota*, which accounted for 49.2–72.1% of the total sequences, and other fungal phyla species were <1% of the species abundances (Figure 5b). The relative abundance of *Proteobacteria* was significantly higher in 15-year-old (37.9%) stand than those in 7-year-old (33.1%) and 25-year-old (30.8%) stands of CFP, while *Chloroflexi* in 7-year-old (10.5%) and 25-year-old (9.1%) stands were significantly higher than 15-year-old (5.2%) stands of CFP (*p* < 0.05, Figure 5a). The abundance of *Basidiomycota* was significantly lower in 15-year-old (17.5%) stand of CFP than in 7-year-old (55.6%) and 25-year-old (45.5%) stands, while the abundance of *Ascomycota* and *Rozellomycota* were significantly lower in 7 and 25-year-old stands than in 15-year-old stand of CFP (*p* < 0.05, Figure 5b).

### 2.3. Effects of Soil Abiotic and Biotic Factors on Soil Microbial Community

**Figure 6:**
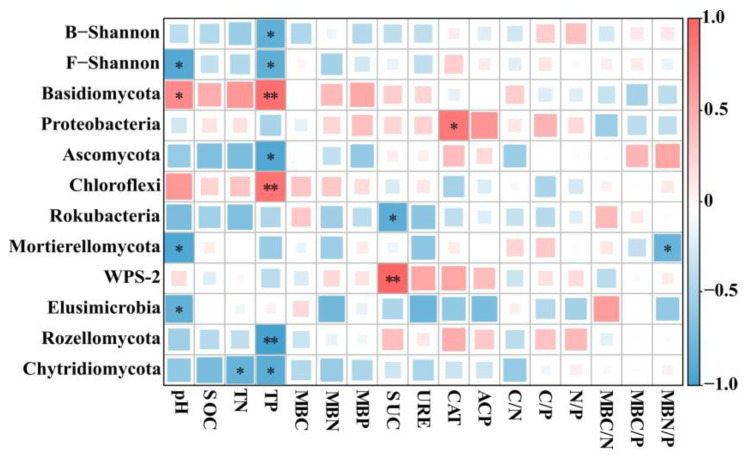
Correlation analysis of soil abiotic factors on microbial diversity and species composition in different stand ages of CFP. * and ** indicate significant at *p* < 0.05 and *p* < 0.01, respectively. SOC is soil organic carbon, TN is soil total nitrogen, TP is soil total phosphorus, MBC is microbial biomass carbon, MBN is microbial biomass nitrogen, MBP is microbial biomass phosphorus, SUC is Sucrase, URE is Urease, ACP is Acid phosphatase, and CAT is Catalase.

Correlation analysis showed that among the soil abiotic factors such as soil nutrients, microbial biomass, and enzyme activity, TP and pH were strongly correlated with microbial diversity and species abundance in different stand ages of CFP, in which, TP content was negatively correlated with bacterial and fungal Shannon index, *Ascomycota*, *Rozellomycota*, and *Chytridiomycota* abundance, and was positively correlated with *Basidiomycota* and *Chloroflexi* abundance (*p* < 0.05, Figure 6). Soil pH was negatively correlated with the fungal Shannon index, *Mortierellomycota*, and *Elusimicrobia* abundance, and was positively correlated with *Basidiomycota* abundance (*p* < 0.05, Figure 6).

**Figure 7:**
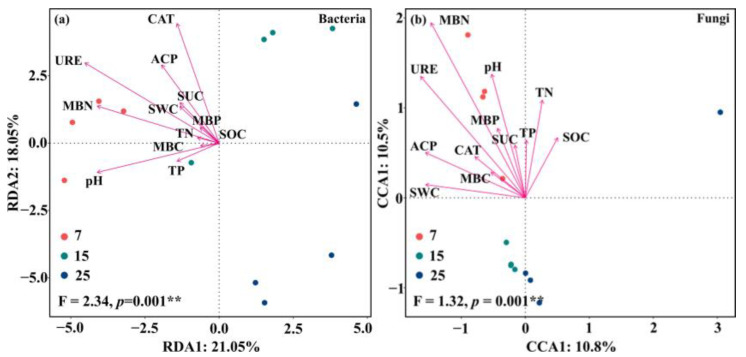
Redundancy and canonical correspondence analysis of the relationships between soil abiotic factors and bacterial (**a**) and fungal (**b**) communities in different stand ages of CFP (7, 15, and 25-year-old). ** indicate significance at *p* < 0.01. SOC is soil organic carbon, TN is soil total nitrogen, TP is soil total phosphorus, MBC is microbial biomass carbon, MBN is microbial biomass nitrogen, MBP is microbial biomass phosphorus, SUC is Sucrase, URE is Urease, ACP is Acid phosphatase, and CAT is Catalase.

Redundancy analysis (RDA) indicated that two standard axes explained 39.1% of the relationship between bacterial species and soil environmental factors (*p* = 0.001, Figure 7a). Among them, soil URE, MBN, pH, and ACP explained 82.2%, 65.7%, 64.5%, and 53.0% of the bacterial community structure, respectively, and URE was the critical factor affecting soil bacterial species composition (*p* = 0.001, Figure 7a). Canonical correspondence analysis (CCA) showed that two standard axes explained 21.3% of the relationship with soil environmental factors in the fungal community (*p* = 0.001, Figure 7b), where soil MBN, URE, and ACP explained 75.5%, 65.6%, and 50.7% of the fungal community structure, respectively, and MBN was the principal factor affecting on soil bacterial species composition (*p* = 0.001, Figure 7b). In summary, soil MBN content, URE, and ACP activities were the main environmental driving factors on microbial species composition in different stand ages of CFP.

## 3. Materials and Methods

### 3.1. Study Site

**Figure 8:**
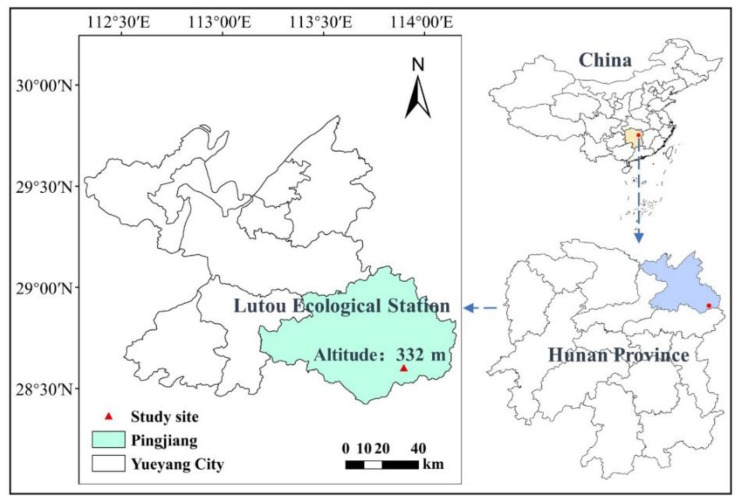
The experimental study sites are in North Luoxiao National Forest Park, Pingjiang County, Yueyang City, Hunan Province of China.

The study site is located in the Lutou Experimental Forest of North Luoxiao National Forest Park, Pingjiang County, Yueyang City, Hunan Province, China (113°53′ E, 28°36′ N) (Figure 8). The landform is low hills with altitudes between 124–1272 m and an average slope is 30°. The study area has a typical subtropical humid continental climate with an average annual temperature of 16.8 °C and an average annual precipitation of 1450.8 mm. The soil is a typical mountainous yellow loam with a soil pH value of 4.6–5.0. The forest area of the study site is 4762 hm^2^, the forest coverage rate is 94.2% with a standing volume of 41.2 × 10^4^ m^3^, and the CFP area is 775 hm^2^. The understory shrubs species consisted of *Maesa japonica*, *Smilax china*, and *Camellia japonica*, and the herbs included *Parathelypteris* and *Ophiopogon japonicus* in the CFP stands.

### 3.2. Experimental Design and Soil Sampling

The split-plot design with different stand ages of CFP as the main factor and the soil depth (0–10, 10–20, 20–30, and 30–40 cm) as the sub-factor was used in this study. Four replicated 20 m × 20 m plots were set up in each of the young (7-year-old), middle (15-year-old), and mature (25-year-old) CFP. The stand information of different stand ages of CFP is shown in Table 1. Soil samples were collected in August 2021, which were diagonal taken from five sampling points in each plot at the soil depth of 0–10, 10–20, 20–30, and 30–40 cm, respectively. A total of 48 soil samples (3 stand ages × 4 soil depth × 4 replication) were corrected in the study sites. All soil samples were transported to the laboratory in ice boxes and then passed through a 2 mm sieve to remove the roots, litter, and stones. One subsample (approximately 10 g fresh soils) was stored at −80 °C for DNA extraction. A portion of soil samples (approximately 200 g fresh soil) was stored at 4 °C for the determination of soil microbial biomass carbon (MBC), nitrogen (MBN), phosphorus (MBP), and enzyme activities. Another portion of soil samples was air-dried and then sieved with a 0.149 mm sieve for measuring the soil pH, soil organic carbon (SOC), total nitrogen (TN), and total phosphorus (TP).

### 3.3. Soil Sample Analysis

Soil pH was measured by a PXS-270 ion meter, with a soil/water ratio of 1:2.5 (*w*/*v*). Soil moisture content (SWC) and soil bulk density (SBD) were determined by drying fresh soil to constant weight at 105 °C [21]. SOC was measured by the K_2_Cr_2_O_7_-H_2_SO_4_ oxidation method. TN was determined by the micro Kjeldahl and steam distillation methods. TP was measured by the molybdenum antimony colorimetric method [7]. Soil MBC, MBN, and MBP were analyzed using the trichloromethane fumigation culture method [13]. Acid phosphatase (ACP) activity was determined by the p-nitrophenyl phosphate disodium (PNPP) method. Urease (URE) activity was analyzed using the sodium phenol-sodium hypochlorite colorimetric method. Sucrase (SUC) activity was measured by the 3,5-dinitro salicylic acid colorimetric method. Catalase (CAT) activity was determined by the potassium permanganate titration method [13]. Soil microbial community β-diversity was determined using nonmetric multidimensional scaling (NMDS) analysis at the ASV level [16]. Soil physicochemical properties in different stand ages of CFP are shown in Table 1.

### 3.4. DNA Extraction and High-Throughput Sequencing

Extract total soil genomic DNA from 0.25 g of fresh soil sample using the E.Z.N.A. Follow the manufacturer’s instructions with a soil DNA isolation kit (Omega, Bio-Tek, Norcross, GA, USA). DNA was extracted three times from each soil sample, then subsequently pooled and quantified using a spectrophotometer (NanoDrop2000, Technologies, Wilmington, DE, USA). All extracted DNA was stored at −80 °C until PCR amplification and sequencing of the 16S rRNA and internally transcribed spacer (ITS) genes [1].

The target 16S rRNA and ITS gene fragment were amplified using barcode primers set 228F/806R and ITS1F/ITS2, respectively. PCR amplification of the bacterial 16S rRNA gene was performed in a 50 µL mix containing 0.5 µL (20 µM) of each primer, 25 µL (2 U) of Taq DNA polymerase mix, 1 µL (~30 ng) of template DNA, and 23 µL sterile deionized water. PCR reactions were performed with an initial denaturation (95 °C, 3 min), 27 cycles (95 °C, 30 s; 55 °C, 30 s; 72 °C, 30 s), and a final extension (72 °C, 10 min). The fungal ITS PCR amplification was performed in a 20 µL mix containing 0.8 µL (5 µM) of each primer, 4 μL of 5× FastPfu Buffer, 2 μL of 2.5 mM dNTPs, 0.4 μL of FastPfu Polymerase, and 10 ng of template DNA. The ITS PCR reactions were performed with an initial denaturation (94 °C, 2 min), 35 cycles (95 °C, 30 s; 55 °C, 30 s; 72 °C, 60 s), and a final extension (72 °C, 10 min). Amplicons were extracted from 2% agarose gels and purified using an AxyPrep DNA Gel Extraction Kit (Axygen Biosciences, Union City, CA, USA) according to the manufacturer’s instructions and quantified using QuantiFluor™-ST (Promega, Madison, WI, USA). Purified amplicons were pooled in equimolar ratios and paired-end sequenced (2 × 250) bp on an Illumina MiSeq platform according to standard protocols. Raw FASTQ files were demultiplexed and quality-filtered using QIIME (version 1.17) with the criteria [22].

### 3.5. Statistical Analysis

Statistical tests for the effects of stand ages on soil physicochemical properties (pH, SWC, SBD, SOC, TN, and TP), soil microbial biomass (MBC, MBN, and MBP), enzyme activity (ACP, URE, SUC, and CAT), and microbial diversity and community composition were performed by one-way analysis of variance (ANOVA). We calculated sample means and standard errors (SE) of soil environmental factors for CFP at different stand ages and tested whether these indicators were significantly different among 7, 15, and 25-year-old CFPs using the Tukey–Kramer test. The Nonmetric multidimensional scaling analysis was performed on the OTU data using the R package (V.3.4.0) to distinguish microbial community profiles at different stand ages. Pearson correlation analysis, multiple regression analysis, redundancy analysis, and canonical correspondence analysis were used to explore the relationships and interactions between soil environmental factors and microbial communities. Statistical analyses were conducted using R and SPAA 22.0 software.

## 4. Discussion

### 4.1. Variation of Soil Nutrients and Enzyme Activities in Different Stand Ages of CFP

Soil SOC, TN, TP, MBC, MBN, and MBP contents were highest in the young stand of CFP and then significantly decreased in the middle-aged stand, and then recovered significantly in mature stands during the development of CFP in our study. Soil nutrient content is an essential factor influencing soil microbial biomass, plant growth, and nutrient cycles [1]. The early stage of CFP can greatly improve soil health and promote nutrient accumulation in soils [23]. CFP is a fast-growing tree species, the middle stage of CFP is the maximum growth period with strong competition for stands, increased nutrient requirements, and massive shifts of C, N, and P from soil to plants [24]. However, the increase in forest canopy density and the decrease in light led to the decrease of understory vegetation and soil organic matter, which significantly decreased the content of C, N, and P [6]. In the mature stage of CFP, its slow growth reduces the demand for nutrients, and plant residues return nutrients, increasing the content of C, N, and P in the soil [25]. Consistent with the previous findings, soil nutrient contents showed a decreasing trend with deeper soil layers in different stand ages of CFP, which was attributed to surface soil being the main place for nutrient transformation and accumulation by microorganisms.

Soil C:N:P stoichiometry not only reflects soil fertility and plant nutrient profiles, but also serves as a diagnostic and predictive indicator of nutrient limitation and C, N, and P saturation [26]. In this study, the ratios of soil C:N and C:P in the three stand ages of CFP were lower than the average values of Chinese plantation soils, while the opposite was for the N:P ratio. The phenomenon of imbalance of soil C, N, and P stoichiometry in CFP soil could be explained that due to slow decomposition rates of litterfall in the topsoil layer of CFP, which is not conducive to the release of organic matter, besides there exists a serious phosphorus deficiency in Chinese subtropical forest soils [27,28].

The four enzyme activities (SUC, URE, ACP, and CAT) of soil significantly decreased with increasing stand ages and the soil’s enzyme activities and nutrient contents were highest in the young stands in this study. Although soil nutrients were recovered in mature stands, soil enzyme activities were the lowest. Numerous studies have shown that soil URE, ACP, and SUC are all intimately related to soil quality in plantation forests, and their activities can rapidly respond to soil nutrient changes [9]. When CFP enters the mature stage, changes in microclimatic conditions (such as temperature and sunlight) in the stand will reduce soil microbial metabolism, resulting in a decrease in soil enzyme activity [29].

### 4.2. Variations of Soil Microbial Diversity and Composition in Different Stand Ages of CFP

Forest type and soil properties largely determine soil microbial diversity [20]. The alpha diversity of soil bacterial and fungal communities was constantly higher in 15 and 25-year-old stands than in 7-year-old stand in our study. Many studies have shown that with increasing stand age, the species diversity of understory vegetation increases, and the large accumulation of humus is conducive to the growth of microbial populations [1,30]. In addition, the degrees of soil nutrient consumption and utilization differed at different developmental stages of CFP leading to significant differences in microbial diversity and composition. Soil environmental factors were shown to be the most important factors driving soil bacterial community diversity compared with vegetation factors soil environmental factors proved to be the most important factors driving soil bacterial community diversity compared with vegetation factors [31,32]. In this study, there was no significant difference in soil bacterial community diversity among 7, 15, and 25-year-old stands of CFP could be attributed to soil nutrient limitation in middle-aged development. Due to the physiological and ecological characteristics of microorganisms, soil fungal and bacterial communities have great differences in adaptation to the environment, a considerable number of soil fungal communities exist in the form of plant-mycorrhizal symbiosis, and host plants strongly influence fungal communities through the formation of specific understory litter and underground root biotic residues [33,34,35]. However, the above-ground tree and soil taproot networks were dominated by single tree species in CFP, which may lead to insignificant differences in soil fungal community diversity in different developmental stages of CFP [13].

In addition, the NMDS analysis revealed that the soil bacterial and fungal communities were distinctly divided into three components, indicating significant differences among different stand ages. In the soil bacterial community, the *Proteobacteria* and *Acidobacteria* had higher abundance, followed by *Actinobacteria* and *Chloroflexi*, and their abundances varied significantly among different stand ages, which were supported by the previous studies [13]. Among them, *Proteobacteria* is the largest phylum in the classification of known bacterial species. *Acidobacteria* had a high abundance in subtropical acidic yellow-brown soils due to their acidophilic properties. *Actinobacteria* was significantly positively correlated with soil organic matter content, and *Chloroflexi* was a group of bacteria that produce energy through photosynthesis and therefore are more abundant in the young forest stage [6,36]. In the soil fungal community, the most abundant phyla were *Basidiomycota* and *Ascomycota*, and their abundances varied significantly in three stand ages. Studies have shown that the lignocellulosic organic matter content in coniferous forest apoplast is higher than that in broad-leaved forests, and the fungal taxa are mainly distributed in *Basidiomycota* phylum [37]. In addition, fungal species diversity was directly or indirectly affected by soil temperature and humidity, pH, and nutrient content [38]. However, these environmental factors differed significantly in the three stand ages of CFP.

### 4.3. Effect of Soil Environmental Factors on Microbial Communities in CFP

Soil TP and pH were strongly negatively correlated with soil microbial diversity and phylum abundance levels in CFP in the study sites. Previous studies have demonstrated that soil phosphorus is the limiting nutrient for the growth and development of CFP in subtropical regions of China. P is a key component of biological genetic material nucleic acid and cell membrane phospholipids and is also a structural element of ATP, which is a carrier substance of biological cell energy metabolism. The previous studies demonstrated that soil P is a limiting nutrient for the growth and development of CFP in subtropical regions of China [28,39]. Soil pH is capable of affecting the composition, chemical properties, and utilization efficiency of soil substrates, and has been demonstrated as an important abiotic factor driving microbial community variation and spatial differentiation in forest ecosystems [40].

Moreover, the results of RDA and CCA showed that soil MBN content, URE, and ACP activities highly explained the bacterial and fungal species composition. Soil N and P are the two most important nutrients for plant growth. MBN is the most active component of soil organic nitrogen and plays an essential role in regulating soil nitrogen cycling and transformation [41]. Numerous studies have found that nitrogen addition or nitrogen deposition can directly or indirectly alter nitrogen availability and nutrient allocation patterns between soil microbes and plants, thereby affecting microbial diversity and community composition, e.g., nitrogen increase simultaneously decreases soil microbial diversity and *Actinobacteria* relative abundance [42,43]. The function of soil urease involves making urea available to plants by converting it to ammonia, while soil phosphatase is catalyzing soil organic P compounds to be mineralized into inorganic P and directly affect the biological effectiveness of soil P, and urease and phosphatase activities are closely related to soil N and P contents and Microbial species abundance [16,29]. However, soil nutrient contents and enzyme activities were imbalanced with microbial species diversity and abundance with stand age development in this study. Especially, bacteria and fungi almost respond consistently to specific soil environmental factors, overall soil N and P nutrient balance and their associated invertase activities were to be the important factors affecting the evolution of microbial communities, these environmental factors should be taken into account in the future studies on CFP nutrients and microorganisms [44].

## 5. Conclusions

In this study, we found that the stand ages of CFP and soil depth have significant effects on soil nutrients, enzyme activity, and element stoichiometry characteristics. The soil nutrients and enzyme activities had a decreasing pattern in the middle stages of CFP development. In addition, the stand ages significantly influenced the soil bacterial and fungal diversity and community structure. The highest soil nutrient contents and enzyme activities were found in the surface soil layer of the young stage of CFP. A strong correlation between soil TP and pH with microbial diversity and species composition was found. The differences in bacterial communities were caused by soil URE, MBN, and pH, while differences in fungal communities were caused by soil MBN, URE, and ACP. Overall, soil MBN content and URE activity were the major biotic factors affecting the soil microbial community structure in different stand ages of CFP. Our research results imply that forest management practices, such as accelerating the decomposition of litterfall, to improve soil nutrient contents should be implemented during middle-aged development, which will be greatly meaningful for the sustainable and scientific management of CFP.

## Figures and Tables

**Table 1:** Stand information of different stand ages of CFP.

Stand Age (Year)	7	15	25
Overstory			
Mean DBH (cm)	6.5	17.3	20.8
Mean height (m)	4.5	16.3	18
Canopy closure (%)	0.5	0.7	0.85
Shrubs coverage (%)	10%	28%	70%
Herbs coverage (%)	82%	60%	75%
Slope aspect	WS	ES	WS
Gradient (°)	29.6	29.2	45.2
Altitude (m)	438.4	281.3	278
Soil properties			
SWC (%)	28.63 ± 0.96 A	21.50 ± 1.91 B	20.17 ± 1.93 B
SBD (g/cm^3^)	1.08 ± 0.08	1.32 ± 0.04	1.28 ± 0.03
pH	4.96 ± 0.01 A	4.67 ± 0.04 B	4.66 ± 0.04 B
SOC (g/kg)	15.71 ± 1.28 A	9.11 ± 0.93 B	11.59 ± 0.83 B
TN (g/kg)	1.96 ± 0.13	1.58 ± 0.10	1.70 ± 0.06
TP (g/kg)	0.39 ± 0.03 A	0.27 ± 0.01 B	0.34 ± 0.01 A
C/N	7.86 ± 0.29 A	5.55 ± 0.27 B	6.48 ± 0.35 B
C/P	39.84 ± 1.52	33.59 ± 2.95	33.91 ± 2.79
N/P	5.01 ± 0.19	5.85 ± 0.28	5.06 ± 0.26
MBC (mg/kg)	505.52 ± 30.04 A	330.03 ± 15.60 B	409.79 ± 40.60 AB
MBN (mg/kg)	190.44 ± 7.76 A	124.08 ± 9.77 B	101.74 ± 7.60 B
MBP (mg/kg)	7.27 ± 0.83	4.66 ± 0.55	5.12 ± 0.73
MBC/MBN	3.05 ± 0.19 B	2.80 ± 0.23 B	4.43 ± 0.46 A
MBC/MBP	99.03 ± 15.78	77.43 ± 9.96	111.08 ± 18.54
MBN/MBP	31.59 ± 3.56	28.96 ± 3.64	25.35 ± 1.34
MBC/MBN	3.05 ± 0.19 B	2.80 ± 0.23 B	4.43 ± 0.46 A
MBC/MBP	99.03 ± 15.78	77.43 ± 9.96	111.08 ± 18.54
MBN/MBP	31.59 ± 3.56	28.96 ± 3.64	25.35 ± 1.34

DBH is the diameter at breast height, WS is southwest, ES is southeast, and CFP is Chinese fir plantation. All data are presented as the mean ± standard error (*n* = 4). Different uppercase letters represent the significant difference in soil physicochemical properties in different stand ages of CFP (Tukey’s honestly significant difference test, *p* < 0.05). SWC is soil water content, SBD is soil bulk density, SOC is soil organic carbon, TN is soil total nitrogen, TP is soil total phosphorus, MBC is microbial biomass carbon, MBN is microbial biomass nitrogen, MBP is microbial biomass phosphorus, and CFP is Chinese fir plantation.

## Data Availability

All relevant data are within the manuscript.
